# Hybrid robot-assisted gait training for motor function in subacute stroke: a single-blind randomized controlled trial

**DOI:** 10.1186/s12984-022-01076-6

**Published:** 2022-09-14

**Authors:** Yen-Nung Lin, Shih-Wei Huang, Yi-Chun Kuan, Hung-Chou Chen, Wen-Shan Jian, Li-Fong Lin

**Affiliations:** 1 Graduate Institute of Injury Prevention and Control, Taipei Medical University, Taipei, Taiwan; 2 Department of Physical Medicine and Rehabilitation, Wan Fang Hospital, Taipei Medical University, Taipei, Taiwan; 3 Department of Physical Medicine and Rehabilitation, Shuang Ho Hospital, Taipei Medical University, New Taipei City, Taiwan; 4 School of Medicine, College of Medicine, Taipei Medical University, Taipei, Taiwan; 5 Taipei Neuroscience Institute, Taipei Medical University, Taipei, Taiwan; 6 Department of Neurology, Shuang Ho Hospital, Taipei Medical University, New Taipei City, Taiwan; 7 Department of Neurology, School of Medicine, College of Medicine, Taipei Medical University, Taipei, Taiwan; 8 Cochrane Taiwan, Taipei Medical University, Taipei, Taiwan; 9 Center for Evidence-Based Health Care, Shuang Ho Hospital, Taipei Medical University, New Taipei City, Taiwan; 10 Institute of Epidemiology and Preventive Medicine, College of Public Health, National Taiwan University, Taipei, Taiwan; 11 School of Gerontology and Long-Term Care, College of Nursing, Taipei Medical University, Taipei, Taiwan; 12 Research Center for Artificial Intelligence in Medicine, Taipei Medical University, Taipei, Taiwan; 13 School of Health Care Administration, Taipei Medical University, Taipei, Taiwan; 14 International Center for Health Information Technology, Taipei Medical University, Taipei, Taiwan; 15 Neuroscience Research Center, Taipei Medical University, Taipei, Taiwan

**Keywords:** Robot-assisted gait training, Stroke, Rehabilitation, Balance

## Abstract

**Background:**

Robot-assisted gait training (RAGT) is a practical treatment that can complement conventional rehabilitation by providing high-intensity repetitive training for patients with stroke. RAGT systems are usually either of the end-effector or exoskeleton types. We developed a novel hybrid RAGT system that leverages the advantages of both types.

**Objective:**

This single-blind randomized controlled trial evaluated the beneficial effects of the novel RAGT system both immediately after the intervention and at the 3-month follow-up in nonambulatory patients with subacute stroke.

**Methods:**

We recruited 40 patients with subacute stroke who were equally randomized to receive conventional rehabilitation either alone or with the addition of 15 RAGT sessions. We assessed lower-extremity motor function, balance, and gait performance by using the following tools: active range of motion (AROM), manual muscle test (MMT), the Fugl–Meyer Assessment (FMA) lower-extremity subscale (FMA-LE) and total (FMA-total), Postural Assessment Scale for Stroke (PASS), Berg Balance Scale (BBS), Tinetti Performance-Oriented Mobility Assessment (POMA) balance and gait subscores, and the 3-m and 6-m walking speed and Timed Up and Go (TUG) tests. These measurements were performed before and after the intervention and at the 3-month follow-up.

**Results:**

Both groups demonstrated significant within-group changes in the AROM, MMT, FMA-LE, FMA-total, PASS, BBS, POMA, TUG, and 3-m and 6-m walking speed tests before and after intervention and at the 3-month follow-up (*p* < 0.05). The RAGT group significantly outperformed the control group only in the FMA-LE (*p* = 0.014) and total (*p* = 0.002) assessments.

**Conclusion:**

Although the novel hybrid RAGT is effective, strong evidence supporting its clinical effectiveness relative to controls in those with substantial leg dysfunction after stroke remains elusive.

*Trial registration* The study was registered with an International Standard Randomized Controlled Trial Number, ISRCTN, ISRCTN15088682. Registered retrospectively on September 16, 2016, at https://www.isrctn.com/ISRCTN15088682

## Introduction

### Epidemiology in stroke

Stroke remains a leading cause of mortality and morbidity globally [1]. Functional disabilities caused by motor impairment are the most common problems after stroke. Approximately 60% of patients lose their ability to walk immediately after stroke [2], and 20% remain unable to walk independently 1 year later [3]. Walking ability in patients with stroke directly affects their quality of life; thus, restoring this ability is a critical goal of rehabilitation.

### Robotic-assisted gait training

Conventional physical and occupational therapy programs have facilitated poststroke neurological and functional recovery. However, these recoveries are usually unpredictable and suboptimal, warranting the development of new strategies to enhance poststroke recovery. Robotic-assisted gait training (RAGT) was introduced as a novel treatment to improve walking recovery. Most RAGT systems are either of the end-effector (e.g., Gait trainer GI I [4] and the G-EO system [5]) or exoskeleton types (e.g., Lokomat [6]), both of which are commonly used to provide programmable gait training during rehabilitation [7]. In the end-effector-based approach, the feet are positioned on two foot plates, which simulate stance and the swing phase of gait. The exoskeleton-based approach involves the use of an exoskeleton with drives that flex the hip and knees during the swing phase as well as a treadmill to simulate the stance phase. The Lokomat system was reported to be more effective than treadmill gait training in improving walking ability, balance, and balance confidence and restoring symmetrical gait patterns with gait discrepancies in patients with chronic stroke [8]. In a study with a different RAGT system, patients with subacute stroke using the hybrid assistive limb robot suit outperformed the control group in terms of improvements in maximum walking speed [9]. Moreover, RAGT with alternating stepping movements was reported to induce physiological muscle activation patterns and improve the Motricity Index and Medical Research Council scores in patients with subacute stroke and healthy controls [10]. Studies have examined the clinical, technical, and regulatory aspects involved in the proposed classification. Clinicians should be aware of each of these perspectives to understand the possible mechanisms underlying recovery in robotic neurorehabilitation [11].

The development of RAGT is founded on the assumption that a task-specific repetitive approach would support motor learning and facilitate functional recovery [12, 13]. The purpose of RAGT is to enable users to perform high-intensity repetitive tasks and reduce the need for support by physical therapists (PTs); with RAGT, a patient can practice 1000 steps within 30 min, which would not be possible with a PT [4]. Although uncertainties remain regarding its beneficial effects on ambulatory function after stroke, two Cochrane systematic reviews have indicated that patients with stroke who received RAGT in combination with physiotherapy in the first 3 months after stroke were more likely to achieve independent walking than those who did not receive RAGT. Their results indicated that the use of electromechanical-assisted devices in combination with physiotherapy may yield some improvements in the biomechanical parameter of walking velocity (meters per second) but not in walking capacity (meters walked in 6 min). The authors of the two studies recommended more trials to evaluate the device type, training frequency, and duration parameters [7, 14].

### Effects of RAGT in stroke

Several studies have responded to the aforementioned call for further research by examining different types of RAGT (end-effector or exoskeleton), treatment protocol intensities (3–8 weeks), and onset phases (subacute, chronic, and 8 weeks to 6 months) after stroke [15–20]. Studies have indicated that patients in the subacute phase with severe disabilities may benefit the most from progressive reduction in assistance force control, which means increased training load in terms of weight bearing for the lower extremities during RAGT, combined with conventional physiotherapy [17, 18, 20]. Research has yielded inconsistent findings regarding the beneficial effects of RAGT and technology-assisted gait training (TAGT) with body weight support (BWS) in terms of ambulatory function (Functional Ambulation Category), balance [Berg Balance Scale (BBS)], and gait (speed, kinematics); moreover, RAGT and TAGT with BWS have been reported to not be significantly superior to traditional rehabilitation [15, 16, 19].

Several studies have evaluated the effects of RAGT in patients with stroke [21–26]. A Cochrane review indicated that RAGT combined with physiotherapy increased gait velocity and odds of participants being able to walk independently [22]. However, some studies have revealed that the improvement was not superior to that of conventional treatment, even when delivered as an add-on to routine rehabilitation in the subacute phase of stroke [21, 24], whereas others have demonstrated beneficial effects compared with physiotherapy alone on clinical functional outcomes and gait pattern [23, 25, 26]. One multicenter randomized controlled trial (RCT) adopted overground RAGT (o-RAGT) and used the Wearable Powered Exoskeleton for treating patients with subacute stroke and helping them walk over ground. Both the o-RAGT and control groups exhibited clinical improvements as indicated by the 6-min walking test, the Modified Ashworth Scale of the Affected lower Limb (MAS-AL), the Motricity Index of the Affected lower Limb, the Trunk Control Test, Functional Ambulation Classification (FAC), a 10 m walking test, the modified Barthel Index, and the Walking Handicap Scale, with nonsignificant differences between the groups [27]. Our research group previously demonstrated changes in the Fugl–Meyer Assessment (FMA), Postural Assessment Scale for Stroke (PASS), BBS, and Barthel Index (BI) scores after an RAGT intervention, indicating substantial improvements in neurological status, balance, and the activities of daily living [28].

### Purpose of the study

Studies on the clinical effects of different types of RAGT have yielded inconsistent findings and follow-up checks have been rare. We therefore conducted an RCT with a novel hybrid RAGT system and a 3-month follow-up to explore the treatment effects (lower-extremity motor function, balance, and gait) in those with subacute stroke.

## Methods

### Participants

The inclusion criteria were as follows: having a first-ever supratentorial stroke in the past 10–60 days, displaying substantial leg disabilities [e.g., a Brunnstrom stage (BS) of I–III in the paretic leg], and being unable to stand or walk independently even with orthotic support (e.g., an FAC score of 0–1). The exclusion criteria were as follows: exhibiting substantial spasticity over the affected leg, severe osteoarthritis, or walking disabilities before the stroke. All patients were randomly assigned 1:1 in a block of four to the experimental or control group after providing informed consent. The patient inclusion flowchart is presented in Fig. 2. The study protocol was approved by the Joint Institutional Review Board of Taipei Medical University (TMU-JIRB No. N201509027) and was explained to all participants before their participation. The study was registered with the International Standard Randomized Controlled Trial Number registry (Trial ID: ISRCTN15088682).

### Stroke characteristics

Basic participant characteristics, including stroke information and comorbidities, were obtained from a chart review. Information regarding lesion location and stroke type was obtained from brain computed tomography or magnetic resonance imaging (MRI). To assess the participants’ independence in terms of activities of daily living (ADLs) [29] pretreatment, the following assessments were conducted: the National Institutes of Health Stroke Scale (NIHSS), MAS, modified Rankin Scale (MRS), Brunnstrom stage (BS), and BI.

### Device

**Fig. 1:**
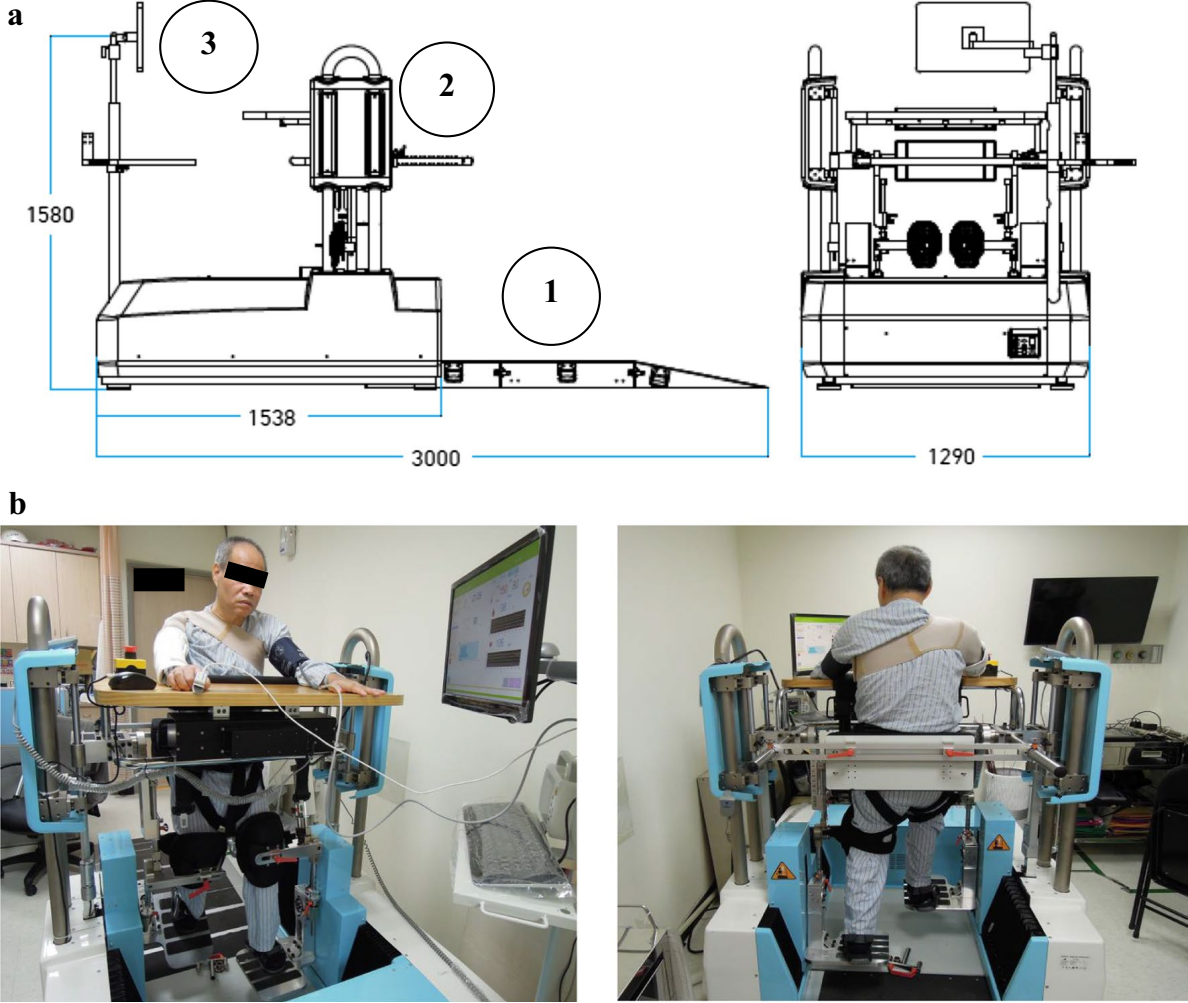
Photographs of frontal and back views of actual use of the Robot Assisted Gait Training (RAGT). **a** Illustrates a dimensional cross-sectional view of the motion mechanism of the RGTS. (1) The transfer system includes a retractable ramp plate and electric body lifting device. (2) The three-point support system (non-suspension system) includes abdominal support, supportive knee caps, and a rear buttock block. (3) The intelligent monitor system (Celeron B810 1.60 GHz 1.88 GB, 32 GB hardware, Microsoft NET Framework 4) includes setting individual user’s basic data, thigh and calf lengths, and training parameters, and monitoring vital signs. **b** Photographs of frontal and back views of actual use of the Robot Assisted Gait Training (RAGT)

The RAGT system (MRG-P100, HIWIN) adopted in this study is a hybrid form of the end-effector and exoskeleton type systems. Both types of devices employ a harness and BWS. However, in the end-effector-based approach, patients’ feet are positioned on two foot plates, whose movements simulate stance and swing phase, whereas in the exoskeleton-based approach, the patient wears an exoskeleton (e.g., Lokomat) with drives flexing the hip and knee during the swing phase, the ankle is passively guided, and a treadmill simulates the stance phase [30]. Our hybrid system consists of a three-point (knees, pelvis, and abdomen) support system, exoskeleton modules and footplates, an electric transfer system, and an intelligent monitoring system (Fig. 1) [28]. The three-point support system (nonsuspension system) provides abdominal support and supportive kneecaps. The transfer system comprises a retractable ramp plate and an electric body lifting device. The peddling cycle is driven by two coordinating footplates and secured by the exoskeleton modules. The intelligent monitoring system (Celeron B810 1.60 GHz 1.88 GB, 32 GB hardware, Microsoft NET Framework 4) enables the input of the individual user’s basic data, thigh (upper leg) and calf (lower leg) lengths, and training parameters and provides vital sign information. When standing on the machine, the patient’s abdomen is secured with the abdominal support from the front. The hip block at the rear prevents the patient from falling backward. The supportive kneecaps from the front avoid knee buckling. In the absence of a suspension harness, which is frequently used in commercial RAGT systems, the three-point support (abdomen, hips, and knees) enables the patient to receive weight-bearing training in a more comfortable environment. The three-point support was designed to help the patient maintain an upright position during gait training. The lack of a suspension harness enables straightforward mounting and dismounting of the system and the performance of intensive training in relative comfort. The development of the desired peddling trajectory was based on the pedal trajectory of an elliptical trainer, and the elliptical-shaped trajectory was demonstrated to have joint kinematics similar to those of a normal walking pattern [31]. The RAGT program predetermines trajectories according to the user’s leg lengths. With a certain leg length, the trajectory can be adjusted automatically using a desired step length, which is presented as the percentage of the maximal step length that is preset in the RAGT program. With a given leg length, the trajectory can be adjusted to different step lengths. In RAGT, step length represents the maximal anterior–posterior displacement of the foot during a peddling cycle. The peddling rate can be set from 1 to 10, corresponding to a walking speed of 0.066–0.917 km/h. The user’s weight is limited to 135 kg. All patients were able to tolerate high-intensity training (e.g., 100% of maximal step length and a peddling rate of 10) through gradual intensity increments. Overall, the system is simple to set up and reduces the need for PT labor, which is consistent with human expectations of robots.

### Intervention

All the patients participated in conventional inpatient rehabilitation programs (five sessions per week, 3–4 weeks, 100 min/session), involving physiotherapy and occupational therapy. The conventional inpatient rehabilitation programs, which involved transfer, sit to stand, static and dynamic balance training, ambulation training, and functional training, were individually tailored to the functional status of each patient. RAGT sessions (30 min each, five sessions per week for 3–4 weeks; total = 15 sessions) conducted by a physical therapist (PT) were added to the conventional inpatient rehabilitation sessions. The 30 min of training included 5 min of warm-up, followed by 20 min of workout, and 5 min of cool-down. The intensity of the warm-up and cool-down was set to 30% of the maximal step length and a speed of 3 for all sessions. The training intensity was elevated by increasing the step length by 10% and peddling rate by 1 for every subsequent session if no discomfort was reported until the maximal intensity (i.e., a ratio of 100% and peddling rate of 10) was achieved. During the sessions, the PT first helped the patient onto the machine (e.g., from sitting in the wheelchair to standing on the footplate of the RAGT), and then input the anthropometric data after measuring the length of the thigh and calf; subsequently, the PT input the training parameters, namely the training duration, step length, and speed, through the intelligent monitoring system. Instead of the RAGT training, those in the control group received an additional 30 min of conventional inpatient rehabilitation (following the same timeline as the experimental group).

### Outcome measurements

We evaluated lower-extremity motor function as the primary outcome and balance and gait performance as the secondary outcome. Lower-extremity motor function was assessed using the active range of motion (AROM), manual muscle test (MMT), and the FMA lower-extremity subscale (FMA-LE) and total score (FMA-total) [32]. Postural control and balance were assessed using PASS [33], Tinetti’s Performance-Oriented Mobility Assessment (POMA) balance and gait subscores, and BBS [34]. The gait performance was assessed using POMA gait subscore, 3-m walk, 6-m walk, Timed Up and Go (TUG) tests, and FAC. The outcome measurements were performed at the pretest, posttest, and 3-month follow-up. In addition, the vital signs (e.g., heart rate, blood pressure, and blood O_2_ saturation) of the patients were monitored during each session. Any discomfort perceived during or after the interventions was recorded.

### Blinding

An independent assessor, who was blinded to the study setup, participant recruitment, and group assignment, assessed all outcomes at an off-site location. The participants were instructed not to discuss their intervention with the assessor or each other.

### Statistical analysis

G*Power (version 3.1.9.2, Heinrich-Heine-Universität, Düsseldorf, Germany) was used to calculate the required sample size. According to a similar study [24], to satisfy an α level of 0.05 and a power of 0.95, a minimum of 20 participants were required in each group. With this sample size, a mean FMA-LE score change of 6 would be necessary to indicate meaningful recovery of lower-extremity function [35]. For between-group comparisons of participant characteristics, the chi-square test was used to compare categorical variables (sex ratio, diabetes mellitus [DM], stroke type, and affected hemisphere), and one-way analysis of variance (ANOVA) was used to compare continuous variables (age, onset time, NIHSS, BS, MRS, BI, and MAS). The within-group and between-group differences at three time points (preintervention, postintervention, and 3-month follow-up) were assessed using repeated-measures ANOVA. The differences between the RAGT group and the control group in terms of AROM, MMT, FMA-LE, PASS, POMA balance subscore, and BBS at the three time points were analyzed using one-way ANOVA and Fisher’s least significant difference (LSD) post hoc test. The POMA gait subscore, 3-m walk, 6-m walk, and TUG results were analyzed using the Mann–Whitney U test and Wilcoxon signed-rank test for within-group and between-group comparisons at two time points (postintervention and 3-month follow-up). Furthermore, we adjusted for the effects of age on postural stability and sensory integration. A chi-square test of independence was used to examine the between-group differences for the categorical variables (FAC) at pretest, posttest, and 3-month follow-up. Specifically, the participants were divided into two subgroups, with 1 and 0 defined as unable to walk and able to walk, respectively. The criterion for entry into the model was set at *p* = 0.05. All statistical analyses were performed using SPSS v19 (IBM, Chicago, IL, USA).

## Results

**Fig. 2:**
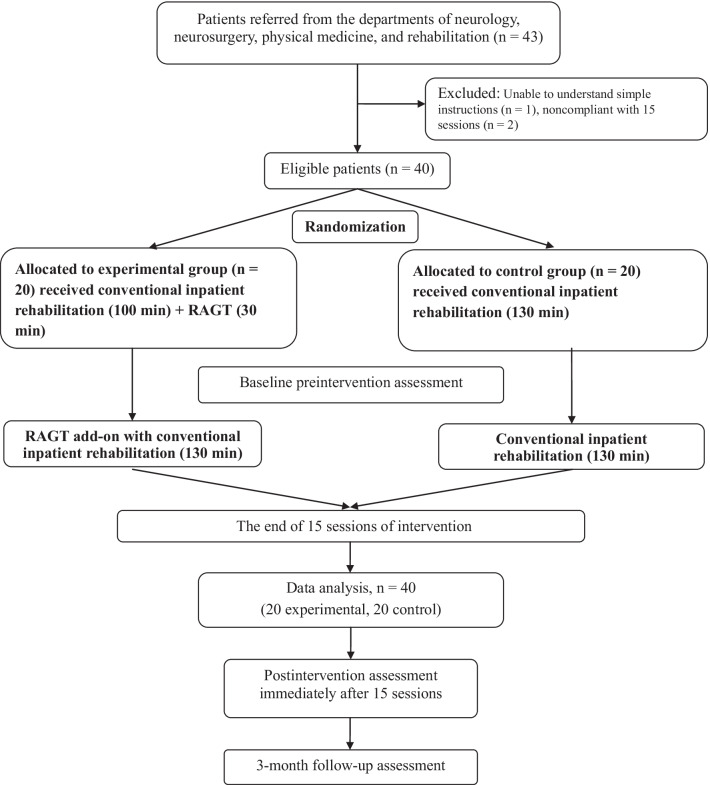
Flowchart depicting patient enrollment

In total, 43 patients with stroke were recruited from the neurological, neurosurgical, and rehabilitation departments of Shuang Ho Hospital. Three of them were excluded because of inability to understand simple instructions (*n* = 1) and inability to complete 15 sessions (*n* = 2). Finally, 40 participants (20 each in the RAGT and control groups) met the eligibility criteria. The flowchart of participant enrollment is presented in Fig. 2. All participants were recruited from an acute stroke care setting approximately 10 days after stroke. Their clinical demographic information is presented in Table 1. All participants were nonambulatory at recruitment, as indicated by the mobility scale and BS in the paretic leg (RAGT: 2.6 ± 1.0, control: 2.8 ± 0.8); thus, all patients exhibited substantial leg paralysis and marked disabilities. The mean age (*p* = 0.50), sex ratio (*p* = 0.50), DM (*p* = 0.50), stroke type (*p* = 0.60), affected hemisphere (*p* = 0.10), onset time (*p* = 0.29), NIHSS (*p* = 0.77), BS (*p* = 0.50), MRS (*p* = 0.50), BI (*p* = 0.50), and MAS (*p* = 0.50) of the patients in the two groups (RAGT and control) did not differ significantly in the pretest (Table 1).

**Table 1:** Participant characteristics in the RAGT group (*n* = 20) and the control group (*n* = 20) at baseline

	RAGT	Control	*P*
Age (years)	54.1 ± 8.6	56.5 ± 12.9	0.50
Sex (female/male)	6/14	5/15	0.50
DM (yes/no)	5/15	2/18	0.20
Type (hemorrhagic/ischemic)	14/6	14/6	0.60
Affected hemisphere (L/R)	13/7	8/12	0.10
Mean onset time (days)	25.8 ± 26.5	34.7 ± 25.5	0.29
NIHSS	12.3 ± 6.0	11.8 ± 5.7	0.77
BS	2.6 ± 1.0	2.8 ± 0.8	0.51
MRS	4.5 ± 0.5	4.5 ± 0.5	1.0
BI	29.3 ± 18.4	33.0 ± 13.8	0.47
MAS	0.2 ± 0.7	0.4 ± .8	0.53

Values are expressed as mean ± standard deviation or number

*DM* diabetes mellitus, *NIHSS* National Institute of Health Stroke Scale, *BS* Brunnstrom stage, *MMT* manual muscle test, *MRS* Modified Rankin Scale, *BI* Barthel Index, *MAS* Modified Ashworth Scale

*P < 0.05 by either chi-square test or independent; *t* test for between-group comparisons

Our results indicate that participants’ ability to perform ADLs improved after RAGT and conventional inpatient rehabilitation. BI at preintervention (RAGT: 29.3 ± 18.4, control: 33.0 ± 13.8), postintervention (RAGT: 47.3 ± 23.3, control: 50.3 ± 22.8), and the 3-month follow-up (RAGT: 75.0 ± 25.4, control: 74.5 ± 23.3) did not differ significantly between the groups (*p* = 0.73). At the 3-month follow-up, we recorded whether the participants continued to receive rehabilitation to examine the persistence of the effects. We noted that 16 participants (80%) in the RAGT group and 17 (85%) in the control group continued to receive rehabilitation treatment at the 3-month follow-up, a nonsignificant difference. BS in the paretic leg increased in both groups, but the between-group difference was not significant at any time point: preintervention (RAGT: 2.6 ± 1.0, control: 2.8 ± 0.8), postintervention (RAGT: 3.3 ± 0.9, control: 3.4 ± 0.7), and 3-month follow-up (RAGT: 3.9 ± 1.0, control: 3.7 ± 0.7) (*p* = 0.24).

The scores for lower limb function, measured using MMT (*p* < 0.001), AROM (*p* < 0.001), FMA-LE (*p* < 0.001), and FMA-total (*p* < 0.001) improved significantly in both the RAGT and control groups after the 15-session treatment (Table 2). MMT (*p* = 0.645) and AROM (*p* = 0.873) scores were not significantly different between the RAGT and control groups after the main intervention or at the 3-month follow-up. Notably, the FMA-LE (*p* = 0.014) and FMA-total (*p* = 0.002) scores were significantly higher in the RAGT group than in the control group before and after the 15-session intervention and at the 3-month follow-up. The higher FMA-LE scores for those in the RAGT group (preintervention: 7.0 ± 4.7, postintervention: 12.5 ± 5.8, follow-up: 16.5 ± 7.2) indicated that they obtained a greater improvement in lower limb motor function than the controls (preintervention: 8.1 ± 4.9, postintervention: 11.5 ± 6.3, follow-up: 13.2 ± 5.9). The higher FMA-total scores for those in the RAGT group (preintervention: 11.4 ± 5.4, postintervention: 17.3 ± 6.9, follow-up: 21.4 ± 8.1) indicated that they obtained a greater improvement in lower limb motor function (including coordination function) than the controls (preintervention: 11.7 ± 5.7, postintervention: 15.8 ± 7.3, follow-up: 17.8 ± 7.0). The results of the post hoc test (LSD) revealed that the FMA-LE and FMA-total scores did not significantly differ between the RAGT and control groups. The POMA balance subscore, PASS, and BBS significantly improved in both groups. The POMA balance subscore (*p* = 0.562), PASS (*p* = 0.548), and BBS (*p* = 0.394) did not differ significantly between the groups at postintervention or 3-month follow-up. The higher scores for POMA balance, PASS, and BBS suggest improvements in balance function.

**Table 2:** Within-group and between-group comparisons of lower limb motor function and balance in the RAGT group (*n* = 20) and control group (*n* = 20) at preintervention, postintervention, and 3-month follow-up

	RAGT				Control				*P*^b^ value	Cohen’s *d* post	Cohen’s *d* 3-month
	Pre	Post	3-month follow-up	*P*^a^ value	Pre	Post	3-month follow-up	*P*^a^ value			
MMT	1.7 ± 1.3	2.7 ± 1.5	3.5 ± 1.2	< 0.00*	1.8 ± 1.3	2.5 ± 1.4	3.3 ± 1.0	< 0.00*	0.645	0.14	0.18
AROM	25.0 ± 31.0	43.8 ± 35.6	61.8 ± 34.4	< 0.00*	26.5 ± 28.6	41.8 ± 35.1	60.3 ± 27.8	< 0.00*	0.873	0.06	0.05
FMA-LE	7.0 ± 4.7	12.5 ± 5.8	16.5 ± 7.2	< 0.00*	8.1 ± 4.9	11.5 ± 6.3	13.2 ± 5.9	< 0.00*	0.014*	0.17	0.50
FMA-total	11.4 ± 5.4	17.3 ± 6.9	21.4 ± 8.1	< 0.00*	11.7 ± 5.7	15.8 ± 7.3	17.8 ± 7.0	< 0.00*	0.002*	0.21	0.48
POMA balance subscore	3.2 ± 3.5	8.1 ± 5.6	12.1 ± 4.6	< 0.00*	3.9 ± 3.7	7.9 ± 5.8	11.6 ± 4.8	< 0.00*	0.562	0.04	0.11
PASS	17.1 ± 7.3	25.4 ± 8.3	31.2 ± 5.7	< 0.00*	17.3 ± 9.3	23.6 ± 9.6	30.5 ± 6.7	< 0.00*	0.548	0.20	0.11
BBS	8.2 ± 8.4	22.8 ± 16.7	38.6 ± 15.5	< 0.00*	8.8 ± 9.7	22.4 ± 17.8	34.5 ± 17.0	< 0.00*	0.394	0.02	0.25

Values are expressed as mean ± standard deviation

*MMT* manual muscle test, *AROM* active range of motion, *FMA-LE* Fugl–Meyer Assessment of Lower Extremity (Hip/Knee/Ankle), *FMA-total* Fugl–Meyer Assessment of Lower Extremity total (Hip/Knee/Ankle, coordination/speed), *POMA* balance subscore, Tinetti Performance-Oriented Mobility Assessment Balance, *PASS* Postural Assessment Scale for Stroke, *BBS* Berg Balance Scale

*P < 0.05 by repeated-measures ANOVA for the within-group and between-group comparisons

^a^Within-group difference at preintervention and postintervention

^b^Between-group difference at preintervention, postintervention, and 3-month follow-up

The POMA gait subscore and scores for the 3-m walking, 6-m walking, and TUG tests improved significantly in both groups from postintervention to the 3-month follow-up, except for the POMA gait subscore in the control group at 3 months (Table 3). The POMA gait subscore (*p* = 0.44), 3-m walking test (*p* = 0.88), 6-m walking test (*p* = 0.72), and TUG test (*p* = 0.72) scores did not differ significantly between the groups at postintervention or between postintervention and the 3-month follow-up (*p* = 0.06, 0.57, 0.74, and 0.65, respectively). The POMA gait subscore increased from postintervention to the 3-month follow-up in the RAGT group but decreased in the control group, and the between-group difference neared the level of significance (*p* = 0.066). Notably, higher POMA gait subscores indicate improved gait performance, whereas lower values in the 3-m walking, 6-m walking, and TUG tests indicate improved gait velocity and agility. All participants were nonambulatory at preintervention. At postintervention, one and three patients in the RAGT and control groups, respectively, could walk independently (*p* = 0.29), and seven and five patients in the two groups could walk with an assistive device (*p* = 0.49). Thus, in both the RAGT and control groups, eight patients were able to walk postintervention (*p* = 0.74). At the 3-month follow-up, seven and four patients in the RAGT and control groups, respectively, could walk independently (*p* = 0.29), and eight and twelve patients in the two groups, respectively, could walk with an assistive device (*p* = 0.21); thus, at this time point, 15 and 16 patients in the RAGT and control groups, respectively, could walk (*p* = 0.71). However, the between-group difference in these numbers was not significant at any time point (Table 4).

**Table 3:** Within-group and between-group comparisons of gait performance in the RAGT and control groups at postintervention and 3-month follow-up

	RAGT			Control			*P*^b^ value	*P*^c^ value	Cohen’s *d* post	Cohen’s *d* 3-month
	Post (*n* = 8)	3-month follow-up (*n* = 15)	*P*^a^ value	Post (*n* = 8)	3-month follow-up (*n* = 16)	*P*^a^ value				
POMA_Gait	7.4 ± 2.1	8.4 ± 2.8	0.02*	7.1 ± 3.1	6.6 ± 3.2	0.11	0.44	0.06	0.11	0.60
3 m (s)	17.2 ± 11.9	12.9 ± 13.7	0.01*	14.8 ± 8.0	12.7 ± 8.4	0.02*	0.88	0.57	0.24	0.02
10 m (s)	52.2 ± 36.4	41.5 ± 46.3	0.04*	41.9 ± 22.5	37.9 ± 25.9	0.02*	0.72	0.74	0.34	0.10
TUG (s)	48.7 ± 32.6	35.3 ± 27.1	0.03*	39.4 ± 19.3	37.1 ± 22.8	0.02*	0.72	0.65	0.35	− 0.07

Values are expressed as mean ± standard deviation

POMA_Gait: Tinetti Performance-Oriented Mobility Assessment Gait; 3 m: 3-m walk test; 10 m: 10-m walk test; TUG: Timed Up and Go

*P < 0.05 by *Mann–Whitney* U *Test* and *Wilcoxon* signed-rank *test* for the within-group and between-group comparisons between two time points

^a^Within-group difference at 3-month follow-up

^b^Between-group difference postintervention

^c^Between-group difference at 3-month follow-up

**Table 4:** Participant ambulation status in the RAGT and control groups at postintervention and 3-month follow-up

	Postintervention			3-month follow-up		
	RAGT	Control	*P* value	RAGT	Control	*P* value
Walk independently	1	3	0.29	7	4	0.29
Walk with assistive device	7	5	0.49	8	12	0.21
Walk_overall	8	8	0.74	15	16	0.71

Values are expressed as number of participants. “Walk_overall” is defined as the ability to walk either independently or by using an assistive device

*P < 0.05 by chi-square test for between-group comparisons between two time points (postintervention and 3-month follow-up)

## Discussion

We investigated the effectiveness of a novel 15-session RAGT intervention add-on to a conventional rehabilitation program over 3–4 weeks. To the best of our knowledge, this is the first RCT to apply a hybrid RAGT system (combined end-effectors and exoskeleton) to gait cycle training to improve gait performance. All patients completed the interventions without major problems. Significant within-group improvements were observed in the AROM, MMT, and FMA-LE scores as well as in balance function and gait performance at all time points. However, only the FMA-LE and FMA-total scores were significantly higher in the RAGT group than in the control group. Notably, the POMA gait subscore improved from postintervention to the 3-month follow-up in the RAGT group but regressed in the control group, with the difference nearing the level of significance. Our results indicate that this novel RAGT was beneficial in improving gait performance in patients with severe leg paralysis after stroke, with the effects persisting for 3 months after the intervention.

According to previous studies evaluating the effect of RAGT as an add-on to conventional physiotherapy, patients with subacute or chronic stroke exhibited improved ambulatory function, lower-extremity motor function, balance, and gait performance both immediately postintervention and at follow-up, and these improvements were superior to those in patients receiving conventional physiotherapy [15, 17, 18, 20, 22, 23, 25, 26, 36]. By contrast, some studies have reported that the effects of RAGT in terms of locomotion function, ADLs, and gait were not superior to those of conventional stroke training in patients with subacute stroke [16, 19, 21, 24]. Our RCT focused on the effectiveness of the novel hybrid RAGT system—which enables high-repetition and high-intensity training (step length and gait velocity) with the aim of improving motor function, balance, and gait performance—in patients with stroke both immediately after the 15-session intervention and at the 3-month follow-up. All participants were nonambulatory preintervention. Both immediately after the intervention and at the 3-month follow-up, the number of patients in the RAGT and control groups with improved ambulatory function (independent or assisted walking) was approximately equal. Central drive measurements of participants may thus be a stronger indicator of functional recovery because active participation is the key to successful rehabilitation. The two groups did not differ in terms of improvement in balance and gait performance; this may be because the dynamic postural control and gait adjustment training of those in the control group is a more active approach than passive RAGT. We also observed that the number of participants that improved to independent walking status in the RAGT group (*n* = 7) was higher than that in the control group (*n* = 3), suggesting that RAGT is more effective than conventional training at promoting the recovery of independent activity function at the 3-month follow-up. This beneficial effect of RAGT was also indicated by disparate changes in the POMA gait subscore from postintervention to 3-month follow-up in the RAGT and control groups. The balance component (PASS and BBS) and motor function component (FMA-LE) performance of the participants in previous studies was lower than that of our study participants, which may have influenced the extent of progress after RAGT in our study.

The two types of RAGT systems have specific advantages and disadvantages. Current end-effector systems, which involve suspending the body, allow for a high degree of spatial freedom of movement of the legs, resulting in an unsecured trajectory. Patients with stroke who have poor control of the paretic leg (e.g., poor activation of the knee extensor or unintentional hip rotation caused by spasticity) typically require assistance from a PT to help control the knee or adjust the leg and trunk position to secure the gait trajectory [4, 5]. By contrast, in exoskeleton RAGT systems, the desired gait trajectory can be controlled and automation allows for high-intensity repetitive training in safe conditions, while also reducing the need for PT. However, because of the complex design of commercial exoskeleton-type systems, setting up the system may be challenging. Our pilot study revealed that the proposed RAGT system is feasible, safe, and beneficial when used with nonambulatory patients who exhibit substantial leg dysfunction following stroke. Unlike in other types of commercial RAGT products, in our hybrid system, the gait trajectory is driven by the end-effector and secured by the exoskeleton. This hybrid RAGT system thus leverages the advantages of both system types. Our system was designed for use in patients who exhibit severe leg dysfunction and insufficient active control of the paretic leg, which would hamper conventional standing or ambulation training. This feature may have led to the provision of excessive support, resulting in a lower degree of biomechanical freedom. That is, the participants probably had limited opportunity to challenge their postural control, thereby somewhat diminishing the training benefits.

Although RAGT provides passive gait training, PTs can encourage active participation by the patient in the form of active contraction of the leg muscles (e.g., quadriceps, iliopsoas, and gluteal muscles) during the gait cycles. During passive gait training, the proprioceptive receptors at the joints (e.g., tendons and ligaments) transmit sensory information regarding the weight-bearing position to the cerebral sensory cortex through complex neural connections; in one study, proprioceptive activity was significantly higher in weight-bearing positions than in non–weight-bearing positions [37]. Thus, RAGT can be regarded as a sensory stimulation intervention that may affect neural plasticity after a stroke; this approach has been adopted with the aim of enhancing motor recovery after a stroke [38–44]. Although the sensory and motor systems function differently, these two components are closely linked. For example, sensory feedback is required to properly control body movements, especially during tasks requiring proficiency and dexterity. Moreover, somatosensory deficits can influence motor learning, with poorer motor recovery occurring in those with more severe sensory loss following stroke [45, 46]. Animal and human studies have revealed that sensory input may be connected to brain plasticity and affect the corticomotor excitability of the target area [38, 39, 43, 47–50]. Transcranial magnetic stimulation studies have reported that the excitability of the motor cortex can be increased by applying sensory electrical stimulation to the corresponding body region on the contralateral side or reduced by depriving the area of sensory inputs from the contralateral extremity [39, 41, 48, 51]. With respect to proprioceptive modalities, research on both functional MRI and transcranial magnetic stimulation has indicated that sessions of continuous passive motion of a joint affect cortical excitability [42, 52]. These neurophysiological findings should encourage further clinical trials to explore the clinical effects of RAGT as a proprioceptive intervention on functional performance. In one study, virtual reality–augmented RAGT resulted in high acceptability and motivation, reduced drop-out rate, and extended training time compared with standard RAGT [53].

### Study limitations

First, because of the lack of positive results, this study provides limited support for the effectiveness of the RAGT, but several issues warrant mentioning. For example, the initial level of disability of the participants in this study may have affected the extent of their improvements in the balance component (PASS and BBS) and motor function component (FMA-LE) after RAGT; future research could include participants with more severe disabilities. Moreover, the selection criteria of patients with stroke (e.g., patients with subacute stroke with severe functional impairments) should be carefully considered for RAGT treatment. Second, other essential technical aspects (artificial intelligence–based feedback, step length, and peddle velocity) and training doses (frequency, duration, and session number) should be considered in future studies.

### Implications for clinical practice

This study investigated the effectiveness of a novel hybrid RAGT system as an add-on to a routine inpatient rehabilitation program with the aim of improving lower-extremity motor function, balance, and gait following subacute stroke. Whether this system supports neuromotor recovery of the paretic leg requires further evaluation. Our results demonstrated that RAGT is suitable and beneficial for patients with subacute stroke and substantial leg dysfunction.

### Implications for research

In this study, central drive was difficult to quantify; thus, future studies can measure muscle activation and energy expenditure during RAGT as a proxy for training intensity. Future studies on RAGT should include electromyography of the bilateral lower extremities to maximize the reliability of the findings. Although our results are not particularly satisfactory, our findings, including those regarding the dose and technical concerns, may be valuable for future studies. However, given our evidence that RAGT is a practical treatment solution, our results may encourage future RCTs to explore the effectiveness of RAGT combined with brain stimulation or VR augmentation.

## Conclusions

This study demonstrated that the novel hybrid RAGT add-on inpatient rehabilitation was not superior to conventional intervention immediately after and at the 3-month follow-up after subacute stroke. However, we found some evidence of a beneficial effect (FMA-LE scores) of RAGT on lower-extremity motor function.

## Data Availability

The data sets used and/or analyzed during the current study are available from the corresponding author on reasonable request.

## Abbreviations

RAGT: Robot-assisted gait training
RCT: Randomized controlled trial
AROM: Active range of motion
MMT: Manual muscle test
FMA: Fugl–Meyer Assessment
FMA-LE: Lower-Extremity subscale
PASS: Postural Assessment Scale for Stroke
BBS: Berg Balance Scale
POMA: Tinetti Performance-Oriented Mobility Assessment
TUG: Timed Up and Go
TAGT: Technology-assisted gait training
BI: Barthel Index
BS: Brunnstrom stage
ANOVA: Analysis of variance
